# Digitisation of historical specimens from the Hog Island Audubon Camp Natural History Collection, Maine

**DOI:** 10.3897/BDJ.14.e182222

**Published:** 2026-01-19

**Authors:** Stephen C. Mason, Jr., Scott Weidensaul, Alice Dougherty, Aidan J. Doyle, Mary V. Rady, Liam H. Semmler, Mary A. Steinbicker, Maria T. Mick, Eva M. Lark, Eric Snyder, Vaughn M. Shirey

**Affiliations:** 1 Department of Natural Sciences, Immaculata University, Immaculata, United States of America Department of Natural Sciences, Immaculata University Immaculata United States of America; 2 Hog Island Audubon Camp, National Audubon Society, Bremen, United States of America Hog Island Audubon Camp, National Audubon Society Bremen United States of America; 3 MG Exotic Veterinary Service LLC, Philadelphia, United States of America MG Exotic Veterinary Service LLC Philadelphia United States of America; 4 Department of Biology, Georgetown University, Washington, DC, United States of America Department of Biology, Georgetown University Washington, DC United States of America; 5 Department of Biological Sciences, Marine and Environmental Biology Section, University of Southern California, Los Angeles, United States of America Department of Biological Sciences, Marine and Environmental Biology Section, University of Southern California Los Angeles United States of America; 6 Florida Museum of Natural History, University of Florida, Gainesville, United States of America Florida Museum of Natural History, University of Florida Gainesville United States of America

**Keywords:** Audubon Society, biodiversity, conservation, digitisation, historical specimens, natural history collections

## Abstract

**Background:**

Natural history collections serve as invaluable records of biological complexity across time and space. However, only a small fraction of these collections has been digitised globally, leaving the majority of specimen data inaccessible for research and digital analysis. Hog Island Audubon Camp, located in Bremen, Maine, United States, is a nature and birding education centre operated by the National Audubon Society’s Seabird Institute. It houses a small, but historically valuable natural history collection, primarily used for science education and outreach programmes. By digitising these data, we have increased the research value and accessibility of the collection by enabling scientists to use these records in support of ecological, evolutionary and conservation goals.

**New information:**

Between 2022 and 2024, we digitised all specimens in the collection with available occurrence data using Darwin Core (DwC) as our digital standard. In total, 764 records were captured, representing 417 unique scientific names across animal, plant, fungal and chromista specimens. Most specimens originate from the New England region and the tri-state areas of the United States. Notably, over 300 specimens were collected during the 1960s and 1970s, a period when the harmful effects of DDT (dichlorodiphenyltrichloroethane) on humans and wildlife were becoming increasingly recognised. The oldest specimens in the collection are five New World warblers collected between 1872 and 1897.

## Introduction

A considerable body of evidence has documented that we have entered the Sixth Mass Extinction (Ceballos et al. 2015, Ceballos et al. 2017, Cowie et al. 2022, Ceballos and Ehrlich 2023, Finn et al. 2023, Mata‐Guel et al. 2023). Conservative estimates place current extinction rates at tens to hundreds of times faster than the natural background rate, which highlights the magnitude and urgency of the ongoing global biodiversity loss (Pimm et al. 2006, Ceballos et al. 2015, Cowie et al. 2022). This rapid loss of biodiversity has severe consequences for ecosystem function and resilience, ultimately diminishing ecosystem services that human societies are dependent on, such as food provision, water purification, pollination, nutrient cycling and climate regulation (Worm et al. 2006, Mori et al. 2013, Oliver et al. 2015). Importantly, the full extent of what has already been lost remains unclear, not only because of undescribed and undocumented species, but also due to the limited research on ecological networks and evolutionary processes that sustain known species (Hortal et al. 2015, Tekwa et al. 2023). Much of this critical insight can often be found and, sometimes, can *only* be found, within natural history collections, which serve as invaluable records of biological complexity through time and space (Hedrick et al. 2020, Miller et al. 2020, Shultz et al. 2021).

Natural history collections are estimated to house billions of biological specimens that can help address a multitude of biological questions that involve global climate change, biodiversity conservation, habitat loss and evolution (e.g. changes in phenotype and genotype within populations) (Ariño 2010, Holmes et al. 2016, Cobb et al. 2019, Johnson et al. 2023). Particularly with specimens that date from over 150 years, we can now reconstruct historical communities for areas that have since been urbanised and those experiencing other impacts of global change (Lister 2011, Schmitt et al. 2018, Shultz et al. 2021). However, this can only be done through comprehensive and coordinated digitisation efforts that allow for searchability, accessibility, preservation and interaction (Beaman and Cellinese 2012, Popov et al. 2021). Unfortunately, only a very limited portion of natural history collections has been digitised globally, leaving the vast majority of specimen data as inaccessible (i.e. “dark data") for research and digital analysis (Holmes et al. 2016, Cobb et al. 2019, Miller et al. 2020).

The National Audubon Society has accessioned and maintained hundreds of historical animal, plant, fungal and protista specimens, many of which have been used primarily for science education during birding and natural history camps at Hog Island Audubon Camp in Bremen, Maine. Digitisation of this collection is especially important given that small natural history collections are often highly representative of historically significant time periods and undersampled localities, highlighting their scientific value and the pressing need to make their data discoverable. Additionally, the specimens are frequently handled by both educators and workshop participants, conditions that increase the risk of specimen damage or material loss. Although some specimens lack associated occurrence data (i.e. collection date and location), many include key metadata, such as collection locality, date, sex, morphological measurements and observed behaviour. Therefore, the goals of this project are to: (1) digitise all specimens with available occurrence data and (2) upload the resulting data to the Global Biodiversity Information Facility (GBIF). This initiative will enhance Hog Island Audubon Camp’s ability to access the biodiversity housed in its collection. More importantly, it will expand the research value and accessibility of the specimen metadata, enabling scientists to use these data to support ecological, evolutionary and conservation research at both macro and local scales.

## General description

### Purpose

Hog Island’s Audubon Camp mission is to “conserve and restore natural ecosystems, focusing on birds, other wildlife and their habitats for the benefit of humanity and the earth’s biological diversity”. In alignment with this mission, we digitised all specimens in the Audubon Camp’s Natural History Collection between 2022 and 2024. This effort uncovered several historically and scientifically valuable records, including specimens dating back to the late 1800s, hundreds of specimens collected during the 1960s and 1970s when the harmful effects of DDT (dichlorodiphenyltrichloroethane) on humans and wildlife were becoming apparent and numerous records representative of Maine and the tri-state areas. Uploading these digitised records and their associated metadata to the Global Biodiversity Information Facility (GBIF) enhances the collection’s research value by enabling participants of Audubon Camp’s educational programmes to engage directly with specimen data and improving accessibility for scientists worldwide conducting biodiversity, ecological and conservation research (Maldonado et al. 2015, Heberling et al. 2021).

## Sampling methods

### Sampling description

**Figure 1.**
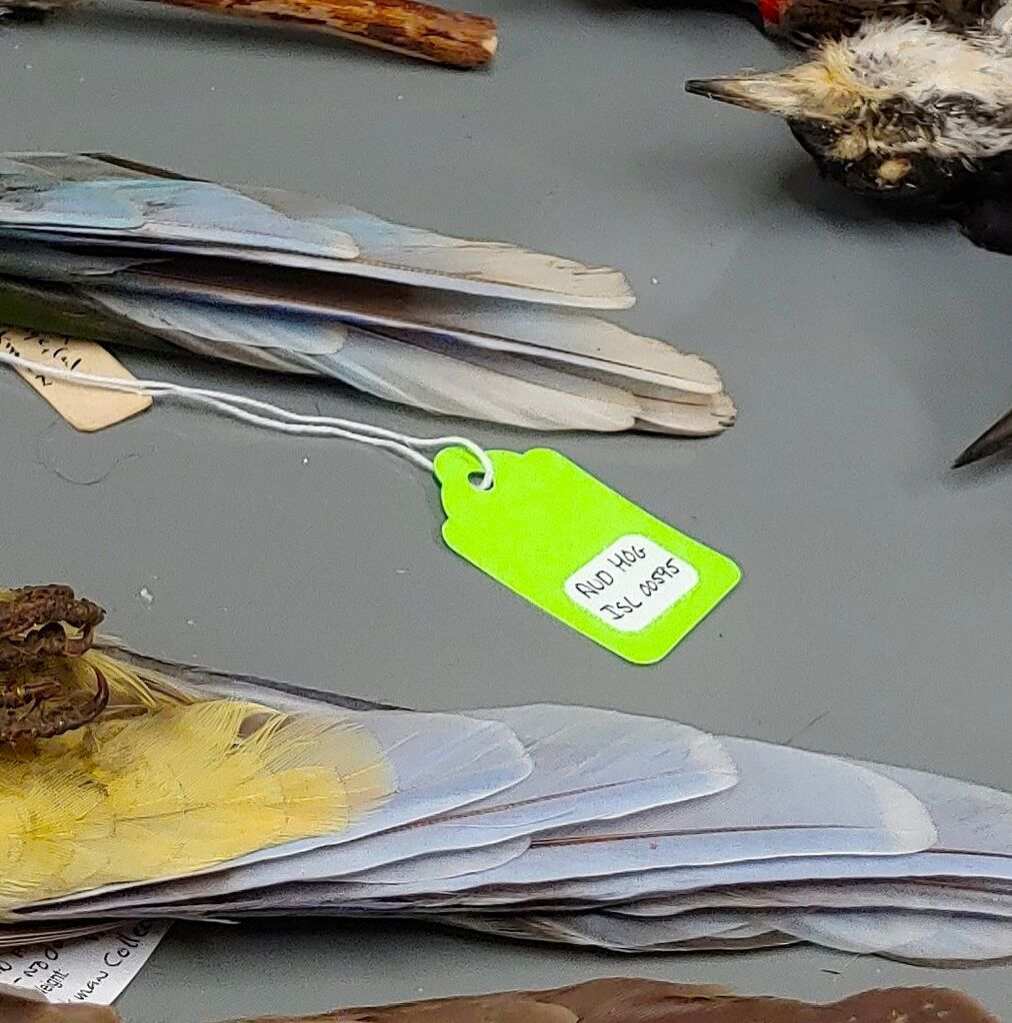
Neon green label on a bird specimen from the Audubon Camp Natural History Collection. These labels indicate that the specimen has been digitised and includes associated occurrence data.

From 2022 to 2024, we digitised the Audubon Camp specimens that contained minimally usable occurrence data (i.e. a taxonomic determination with a location or locality description and/or a year of occurrence). We used Darwin-Core (DwC) as our digital standard for capturing specimen metadata (Wieczorek et al. 2012, Mason et al. 2020). We then performed the digitisation by manually entering the verbatim specimen label data into a shared Google Sheets document. Even if we knew a collector's full name or a misspelling of a species epithet, we kept the verbatim text intact in our Google Sheets document. However, we addressed these discrepancies in our quality assurance check. Each specimen that we finished digitising was assigned a neon green label with a unique identifier using the format “AUDHOGISL#####” (Fig. 1).

Following this transcription of label data, we then used the online platform GeoLocate to assign a decimal longitude, latitude and coordinate uncertainty radius to each occurrence (Seltmann et al. 2018). We used the default uncertainty radii from GeoLocate while conducting this retrospective georeferencing (Seltmann et al. 2018). We performed this georeferencing procedure for all of the localities described below the state level. For example, if a county or parish were provided, we used the county centroid with an uncertainty radius encompassing the entire county. However, if the only locality information provided on the label were a state or province, we did not perform georeferencing. Some verbatim locality descriptions did not return results in GeoLocate, so we did not pursue further georeferencing for those specimens.

### Quality control

During 2023 and 2024, we reviewed the data collected in previous years and identified significant issues that needed to be addressed before making this data more public. This included identifying any misspellings in taxonomic names, as well as an investigation into currently accepted taxonomies. When a new accepted name was identified, we kept the original synonym intact and updated a secondary column with the currently accepted scientific name. We also ensured the proper spelling for data within the “phylum”, “class”, “order”, “family”, “scientificName”, “country” and “stateProvince” columns to the best of our ability.

## Geographic coverage

### Description

**Figure 2.**
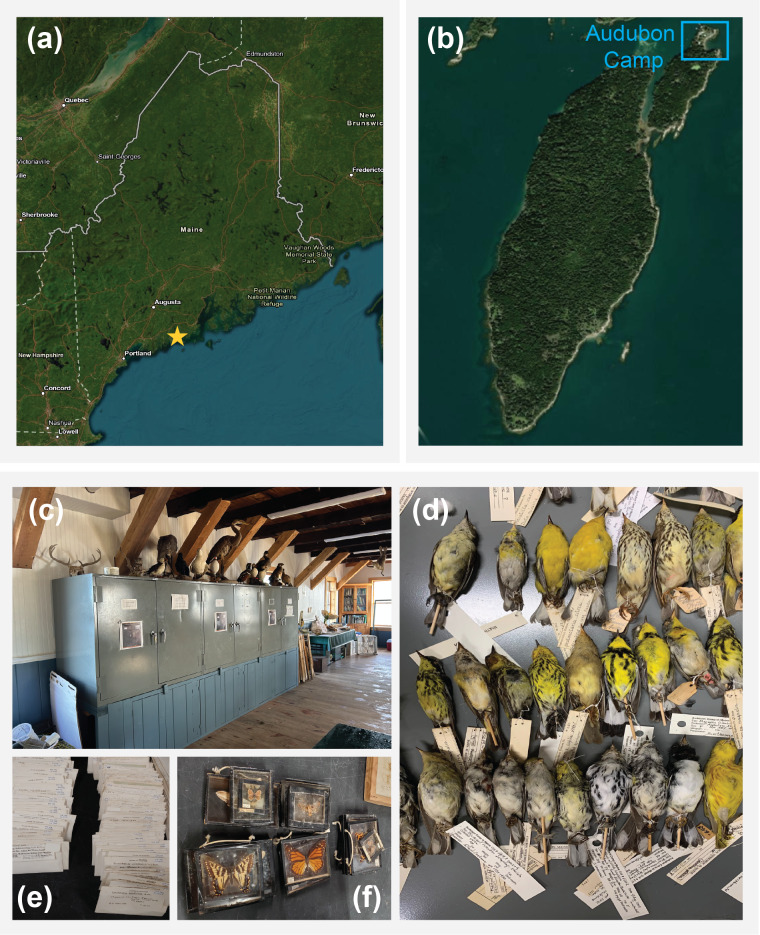
Aerial map of Maine and Hog Island Audubon Camp. The Audubon Camp Natural History Collection consists primarily of vertebrate specimens, but also includes numerous plant, chromista, fungal, insect and other invertebrate specimens.

Hog Island is approximately 330 acres, located in Muscongus Bay, Bremen, ME, with the Audubon Camp located at the northern part of the island (Fig. 2a-b). The Island is predominantly a spruce-pine forest (*Picea rubens* Sargent, 1898, *P. glauca* Sargent and *Pinus strobus* Linnaeus, 1753) with a sparse understorey of mostly spruce and pine saplings, lowbush blueberry (*Vaccinium angustifolium* Aiton, 1789), eastern hayscented fern (*Dennstaedtia punctilobula* Moore, 1857) and bracken fern (*Pteridium aquilinum* Linnaeus, 1753). The northern part of the Island where the Audubon Camp is located includes more broad-leaf trees, such as paper birch (*Betula papyrifera* Marshall, 1785) and oaks (*Quercus* spp.), while a relatively small portion of the southern part of the Island is dominated by common milkweed (*Asclepias syriaca* Linnaeus, 1758) that is located on a former Wabanaki shell heap (Sanger and Sanger 1997, Dawson et al. 2020).

**Figure 3.**
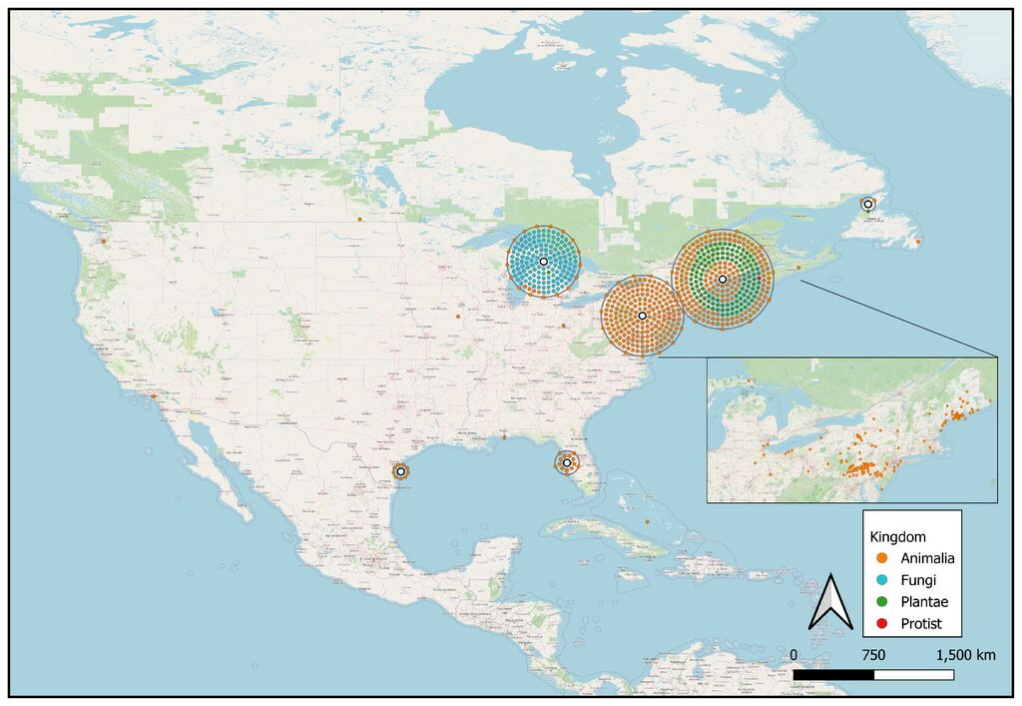
An aerial map illustrating the distribution of new occurrence data digitised in the Audubon Camp Natural History Collection. The larger map displays points aggregated around centroids (white circles) and coloured by taxonomic kingdom. The inset map shows the raw data points for a north-eastern subregion with particularly high volumes of occurrence data.

Most specimens originate from the New England region and tri-state areas of the United States, i.e. Pennsylvania, New Jersey and New York. Specifically, 320 specimens were collected in Maine, 157 in Pennsylvania and a notable cluster of fungi specimens was collected in Michigan. Additionally, 12 specimens were from Canada and one specimen from the Bahamas (Fig. 3). There were 38 specimens for which we did not have georeferenced coordinates.

## Taxonomic coverage

### Description

A total of 764 records were captured, representing 486 animals, 144 fungi, 129 plants and five chromista (Suppl. material 1). These specimens include nine taxonomic phyla; 22 classes; 86 orders; 162 families; 325 genera and 375 species epithets (Table 1). We used the GBIF taxonomic backbone to resolve scientific names. One dubious fungi record labelled as “Asphopulpum pustulatum,” could not be verified, as the genus is unfamiliar and could not be traced to a known determination.

We digitised 417 unique scientific names, though the true diversity within the Audubon Camp’s Natural History Collection is likely higher. This is due for a couple of reasons. First, 41 records were determined only to the genus level or higher, and, second, a few records include more than one specimen, for example, specimens in alcohol vials. In both cases, there are specimens that have not been determined and likely represent species that are not already determined and officially represented in the Audubon Camp Natural History Collection.

A total of 129 unique collector names were recorded (i.e. “recorded by”), after standardising name variants (e.g. S. Kress, Steve Kress, Stephen W. Kress). Amongst the most prolific contributors were author Scott Weidensaul, with 167 bird specimens; M.H. Wells with 96 fungal specimens; Annie Presler, with 39 plant specimens; Shawn Remis, with 38 plant specimens; and Donald J. Borror, with 33 insect specimens (Suppl. material 1).

Approximately 95% of records include latitude and longitude coordinates; 86% include complete collection dates (day, month and year), while 14% include incomplete dates (missing day, month, or year); 78% include the collector (dwc:recordedBy); 41% include dwc:dynamicProperties; 36% include verified sex; 19% include dwc:occurrenceRemarks; 4% include the determiner (dwc:identifiedBy); 2% include dwc:eventRemarks; and 1% include habitat information.

## Temporal coverage

### Notes

**Figure 4.**
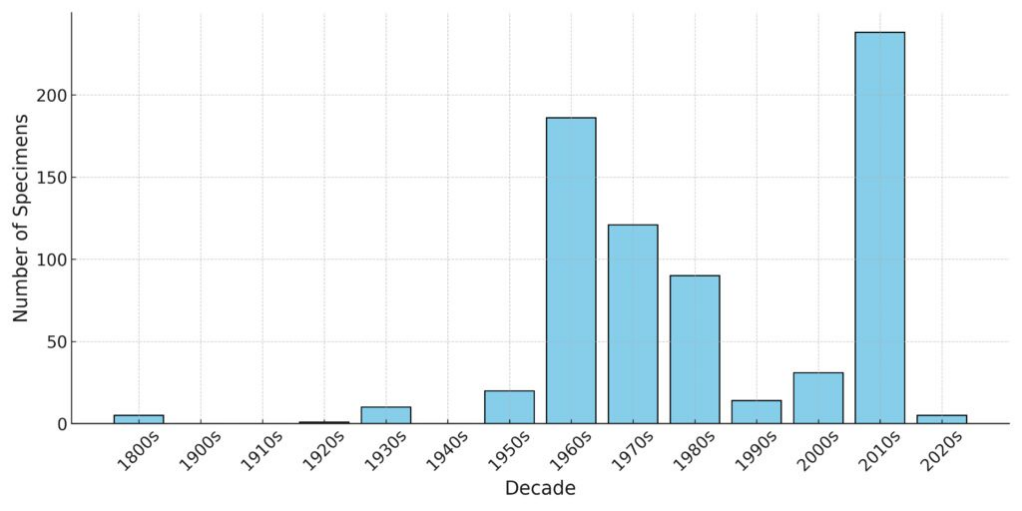
The number of specimens collected by decade in the Audubon Camp Natural History Collection. During the 1960s and 1970s, 306 specimens were collected, with an additional 238 collected in the 2010s. Five of the oldest specimens were collected between 1872 and 1897.

During the 1960s and 1970s, 306 specimens were collected, with an additional 238 collected in the 2010s. The oldest specimens in the collection are five New World warblers collected between 1872 and 1897 (Fig. 4). The New World Warblers are the northern parula (*Setophaga americana*), Canada warbler, (*Cardellina canadensis*), blackburnian warbler (*Setophaga fusca*), chestnuit-sided warbler (*Setophaga pensylvanica*) and blackpoll watrbler (*Setophaga striata*) (Suppl. material 1).

## Collection data

### Collection name

Audubon Camp’s Natural History Collection

### Collection identifier

Specimens are housed in the Hog Island Audubon Camp Natural History Collection and assigned unique identifiers using the format AUDHOGISL#####.

### Parent collection identifier

Specimens were collected and are curated under USFWS Migratory Bird Permit MB70036-A and Maine Department of Inland Fisheries and Wildlife Permit #2022-639.

### Specimen preservation method

Specimens are stored in Lane Science Cabinets; most vertebrate specimens are preserved as skins, wings and tails, while invertebrate, plant, fungal and protist specimens are preserved dry or in vials according to standard natural history collection practices (Figs. 2c-f).

## Usage licence

### Usage licence

Open Data Commons Attribution License

### IP rights notes

The dataset is publicly available at https://doi.org/10.5281/zenodo.18203682 and is licensed under the Open Data Commons Attribution License (ODC-BY), allowing use and reuse with proper attribution.

## Data resources

### Data package title

Historical Specimen Metadata from Hog Island Audubon Camp Natural History Collection

### Number of data sets

1

### Data set 1.

#### Data set name

Supplemental Data 1

#### Data format

.csv

#### Description

This repository contains an .csv file that contains DarwinCore-style metadata associated with specimens in the Hog Island Audubon Camp Natural History Collection in Maine, USA.

**Data set 1.** 

Column label	Column description
institutionCode	Name of institution that holds the specimens.
occurrenceID	Unique identifier for the record.
collectionCode	Code for the specific institution in which the specimens are housed.
catalogNumber	Identifier assigned by the institution to the specimen.
recordNumber	Internal catalogue/record number.
kingdom	Taxonomic kingdom of the specimen.
phylum	Taxonomic phylum of the specimen.
class	Taxonomic class of the specimen.
order	Taxonomic order of the specimen.
family	Taxonomic family of the specimen.
genus	Taxonomic genus of the specimen.
specificEpithet	Species epithet of the specimen.
infraspecificEpithet	Name of subspecies, variety or form of the specimen.
verbatimIdentification	Original determination recorded by collector or observer.
scientificName	Full, accepted scientific name, including infraspecific ranks, using a GBIF backbone.
scientificNameAuthorship	Author(s) of the scientific name.
taxonRank	Taxonomic rank of the identified organism.
day	Day of the month the specimen was collected.
month	Month of specimen collection.
year	Year of specimen collection.
eventDate	Full date of when the specimen was collected.
verbatimLocality	Original, unmodified locality description.
country	Country where the specimen was collected.
stateProvince	State where the specimen was collected.
decimalLatitude	Latitude in decimal degrees.
decimalLongitude	Longitude in decimal degrees.
coordinateUncertaintyInMetres	Estimated spatial uncertainty in metres.
geodeticDatum	Geodetic datum used for coordinates.
georeferenceRemarks	Notes about how coordinates were determined.
sex	Sex of the specimen.
dynamicProperties	Any measurements not captured in other fields.
recordedBy	Name(s) of the collector(s).
identifiedBy	Name(s) of the person(s) who determined the specimen.
habitat	Description of the environment where the specimen was found.
eventRemarks	Additional notes about the collection event.
occurrenceRemarks	Additional notes about the specimen.

## Additional information

Audubon Camp’s Natural History Collection also includes hundreds of specimens that lack associated occurrence data. While these specimens were not digitised, it is important to note that they include historically significant species, such as the extinct passenger pigeon (*Ectopistes migratorius*). We recommend continued educational use of these specimens, despite the absence of occurrence data, as they still hold considerable value for both science communication and learning. Regardless of their data status, all specimens should be handled with care by staff and campers and the collection should continue to be protected as best as possible from environmental stressors (e.g. humidity and sunlight) and biological threats such as carpet or skin beetles (Coleoptera, Dermestidae) (Thacker et al. 2008, Stone and Sikes 2018, Holloway and Bakaloudis 2021). Specimens should remain in a taxonomically curated state to the greatest extent possible, even with their regular use for educational activities. Lastly, capturing high-quality images of the specimens, particularly those with occurrence data, would further support morphometric, colour and structural analyses and other digital analyses that are increasingly valuable in natural history research (Blagoderov et al. 2012, Cope et al. 2012, Borges et al. 2020).

We encourage the continued growth of Audubon Camp’s Natural History Collection through new accessions, research initiatives and contributions from camp programmes and activities. For instance, author Stephen Mason has conducted entomological research with undergraduate students at Immaculata University, resulting in numerous invertebrate specimens, many of which have already been donated to the collection. Similarly, during Family Camp, it is common for instructors to guide campers in pressing and preserving plant specimens collected around the Island. In both cases, associated occurrence data have been recorded, increasing the scientific value of the entomological and botanical materials for the camp to use.

## Supplementary Material

4E9EC5F4-0B22-5C65-AA5F-CB734A5D4568 10.3897/BDJ.14.e182222.suppl1 Supplementary material 1 Historical Specimen Metadata from Hog Island Audubon Camp Natural History Collection Data type Occurrence Brief description The raw historical specimen metadata from Hog Island Audubon Camp Natural History Collection. A total of 764 records were captured, representing 486 animals, 144 fungi, 129 plants and five chromista. The dataset has been submitted to Biodiversity Data Journal and will be publicly accessible via its GBIF endpoint upon manuscript acceptance and dataset publication. File: oo_1509077.csv https://binary.pensoft.net/file/1509077 Mason et al.

## Figures and Tables

**Table 1.** Summary of the abundances of taxonomic Kingdoms, Phyla, Classes, Orders, Families, Genera and species epithets, along with the number of specimens associated with each rank. Species epithets with a lower number than genera are due to having specimens not being determined to the species level. Specimen counts marked with a “>” symbol indicate records of a single species epithet associated with multiple specimens.

**Kingdom**	**Phylum**	**Classes**	**Orders**	**Families**	**Genera**	**Species Epithets**	**Specimens**
Animalia	Arthropoda	2	6	13	34	33	> 54
Animalia	Chordata	3	23	49	120	151	> 430
Plantae	Bryophyta	1	1	1	1	0	1
Plantae	Chlorophyta	2	2	2	3	5	5
Plantae	Marchantiophyta	1	1	1	1	1	1
Plantae	Rhodophyta	2	7	13	14	12	16
Plantae	Tracheophyta	3	27	48	76	73	106
Fungi	Ascomycota	7	16	31	71	110	144
Chromista	Phaeophyta	1	3	4	5	4	5
